# Improved Monitoring of Semi-Continuous Anaerobic Digestion of Sugarcane Waste: Effects of Increasing Organic Loading Rate on Methanogenic Community Dynamics

**DOI:** 10.3390/ijms161023210

**Published:** 2015-09-25

**Authors:** Athaydes Francisco Leite, Leandro Janke, Zuopeng Lv, Hauke Harms, Hans-Hermann Richnow, Marcell Nikolausz

**Affiliations:** 1 Department of Environmental Microbiology, Helmholtz Centre for Environmental Research-UFZ, Permoserstrasse 15, 04318 Leipzig, Germany; E-Mails: athaydes.leite@ufz.de (A.F.L.); zuopeng.lv@ufz.de (Z.L.); hauke.harms@ufz.de (H.H.); 2 Department of Biochemical Conversion, Deutsches Biomasseforschungszentrum Gemeinnützige GmbH, Torgauerstrasse 116, 04347 Leipzig, Germany; E-Mail: leandro.janke@dbfz.de; 3 Department of Isotope Biogeochemistry, Helmholtz Centre for Environmental Research-UFZ, Permoserstrasse 15, 04318 Leipzig, Germany; E-Mail: hans.richnow@ufz.de

**Keywords:** sugarcane waste, biogas reactor overloading, methanogenic pathways, stable isotope fingerprinting, monitoring tool

## Abstract

The anaerobic digestion of filter cake and its co-digestion with bagasse, and the effect of gradual increase of the organic loading rate (OLR) from start-up to overload were investigated. Understanding the influence of environmental and technical parameters on the development of particular methanogenic pathway in the biogas process was an important aim for the prediction and prevention of process failure. The rapid accumulation of volatile organic acids at high OLR of 3.0 to 4.0 g_vs_·L^−1^·day^−1^ indicated strong process inhibition. Methanogenic community dynamics of the reactors was monitored by stable isotope composition of biogas and molecular biological analysis. A potential shift toward the aceticlastic methanogenesis was observed along with the OLR increase under stable reactor operating conditions. Reactor overloading and process failure were indicated by the tendency to return to a predominance of hydrogenotrophic methanogenesis with rising abundances of the orders Methanobacteriales and Methanomicrobiales and drop of the genus *Methanosarcina* abundance.

## 1. Introduction

Sugarcane is widely used in Brazil for bioethanol and sugar production. Bioethanol is well established as fuel primarily in Brazil, whereas sugar supplies the national and international markets. However, the waste generated by the bioethanol/sugar industry consisting mainly of filter cake, vinasse, and bagasse is not managed adequately, since it is still rich in energy when disposed. For the treatment of these waste products, the anaerobic digestion (AD) process was chosen as a promising technology for energy recovery, since the digestate could still be used to fertilize the sugarcane fields, as discussed in our previous study [1,2,3,4].

Despite the methane potential of sugarcane waste, the use of such novel substrates in AD requires research to achieve a process of practical and economic viability. Applying a continuous feeding biogas process along the entire year is a challenge due to the temporal availability of the substrate during approximately 200 operating days of the Brazilian bioethanol/sugar industry. A rapid start-up of the biogas process for more productivity reaching high levels of OLR at the beginning of the operation season may be the solution. Furthermore, the organic loading rate (OLR) is a factor of interest as it determines how much substrate can be treated and converted into biogas per time and reactor volume. However, at high OLR there is a risk of acidification by overloading, potentially followed by process failure. The tightrope walk between exploiting the reactor potential and maintaining a stable process requires cost effective monitoring which allows predicting and assessing process instabilities/failure particularly when the feeding regime is changed. It has been shown that stable isotope fingerprinting of the produced biogas can provide information about the most sensitive functional guild in the AD, the methanogens [5].

In AD the organic matter degradation into biogas proceeds in four major steps, *i.e.*, hydrolysis, acidogenesis, acetogenesis, and methanogenesis, which are carried out by the complex consortia of various bacteria and methanogenic archaea [6]. Methanogenesis, as the terminal phase for methane production, to which two major pathways (aceticlastic and hydrogenotrophic methanogenesis) contribute, is a major target for biogas process optimization [7].

Acetate, H_2_, and CO_2_ (or formate) are the products of the digestion of macromolecules by hydrolytic, acidogenic, and acetogenic bacteria. Aceticlastic methanogens convert acetate into methane and carbon dioxide, whereas hydrogenotrophic methanogens catalyze the conversion of H_2_ and CO_2_ (or formate) to methane. In a stable AD process these complex microbial consortia cooperate and self-regulate their abundances and activities. However, overproduction of organic acids by the bacterial community, e.g., triggered by substrate overload, may overexert the downstream consumption and result in drastic acidification which inhibits the methane production [8]. Thus, to establish and optimize a biogas process with novel substrates such as waste products from the bioethanol/sugar industry, it is essential to study the propensity for acidification and its effect on the methanogenic communities.

Several research studies using different substrates have assessed the microbial diversity in laboratory- and large-scale biogas reactor [9,10,11] and the effect of reactor parameters such as organic acid accumulation on the methanogenic diversity and pathway dominance [12,13,14]. However, to our best knowledge, the effect of acidification by overloading of biogas reactors fed with sugarcane waste has not been investigated. Furthermore, it is known that the susceptibility of AD to overloading depends on the substrate, reactor type, and temperature, thus motivating the present investigation with a novel substrate.

The assessment of the methanogenic community in biogas reactors requires appropriate methods. Variations of the methanogenic community can be analyzed by molecular techniques [15,16], whereas analysis of the biogas isotope composition provides information about the relative contributions of the methanogenic pathways [5,17,18,19]. While molecular biological analyses appear very time-consuming and costly for routine process monitoring, biogas isotope composition appears to be a promising monitoring parameter for industrial biogas processes [20].

Laboratory-scale, semi-continuously-fed, stirred digesters were established to observe the changes of the activity of methanogens as a function of reactor acidification triggered by overloading. Two parallel reactors with mono-digestion of filter cake as substrate, and two other parallel reactors with co-digestion of filter cake and bagasse were monitored by molecular and isotopic techniques to determine the contributions of the methanogenic pathways. Statistical analyses served to correlate isotope signatures and community structures.

## 2. Results and Discussion

### 2.1. Biogas Reactor Performance

In Table 1 the technical parameters and reactor performance during the eight phases of operation are shown. An average value was calculated for each of the two reactor pairs performing mono- and co-digestion. Due to the high volatile organic acids (VOA) concentration (2.45 g·L^−1^) of the digestate mixture used as inoculum, an acclimation of 10 days was required for degradation of the remaining organic matter from the previous reactors. The OLR increased from 0.5 to 4.0 g_vs_·L^−1^·day^−1^ within nine weeks. As a consequence, the hydraulic retention time (HRT) decreased from 36 to 7 and from 37 to 12 days for the mono- and co-digestion reactors, respectively. Along the experiment until Phase 5, the biogas yield was lower for mono- than for co-digestion, whereas the methane content, in mono-digestion reactors was higher. After exceeding the OLR of 2.5 (Phase 5) and 3.0 g_vs_·L^−1^·day^−1^ (Phase 6) for co- and mono-digestion, respectively, the biogas yield decreased drastically. In the co-digestion reactors, the biogas production was inhibited earlier because the pH and the buffering capacity were lowered already in Phase 6. In the following phases, the buffer capacity was insufficient to neutralize the VOA accumulation. The acidification led to decreased biogas yield followed by process failure in both digestion set-ups. Reactor overload and imbalance were already noticed in Phase 6, when the propionate-to-acetate ratios rose from 0.043 to 1.434 (33×) and 0.037 to 1.999 (54×) for mono- and co-digestion, respectively. The results from this phase for mono-digestion were consistent with some other studies, thus confirming that the overload effect is seen earlier from the propionate-to-acetate ratio than from changes in pH or in biogas yield [12,14,21]. Prochazka, *et al.* [22] reported that low ammonium nitrogen (NH_4_-N) concentration (0.5 g·L^−1^) caused low methane yield, loss of biomass and loss of aceticlastic methanogenic activity, and further presented lower buffer capacity and less stable pH. However, this statement does not corroborate our results until Phase 5, indicating that these findings were circumstantial, *i.e.*, depending on the substrate and microbial adaptation. Although the low NH_4_-N concentration during mono-digestion in Phase 6 did not influence negatively the methane yield, foaming was observed in both parallel reactors, which necessitated liquid volume reduction for mono-digestion. The foaming can be ascribed to non-degraded soluble organics, which result in the surface tension reduction of reactor content [23]. At high volatile organic acids per total inorganic carbonate buffer (VOA/TIC) values (3.1 g_VOA_·g_CaCO3_^−1^) in Phase 7, the total- (TS) and volatile solids (VS) values also increased due to the lack of further degradation of the organic matter. This indicated that not just the methanogenesis, but the whole process was inhibited eventually.

### 2.2. Methanogenic Community Dynamics

**Figure 1:**
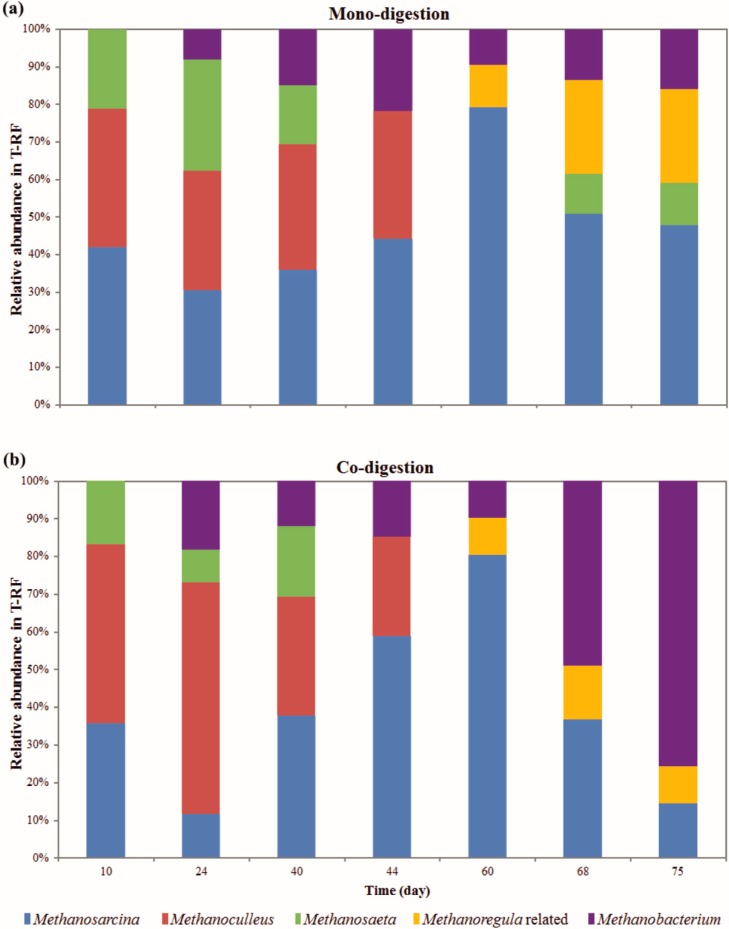
Methanogenic community dynamics in the mono- (**a**) and co-digestion (**b**) reactor. The relative T-RF abundance of methanogens in the digestate samples are given as function of experiment time. For each of the parallel reactors in the specific digestion set-up, two samples were analyzed, that in total four samples were analyzed for each, mono- and co-digestion. All samples belonging to the same digestion set-up had similar methanogenic community based on the relative T-RF abundances. Therefore, each bar on the graphic represents the T-RFLP profile calculated by the average of the four analyzed samples in each digestion set-up. The supporting clone libraries and sequence analysis of the selected clones allowed the taxonomic affiliation of the T-RFs from the community T-RFLP profiles of the complex reactor samples.

The diversity and structure of the methanogenic communities from the mono- and co-digestion were investigated by terminal restriction fragment length polymorphism (T-RFLP) fingerprinting of the *mcrA*/*mrtA* gene (Figure 1) and further validated by sequence analysis of clone libraries (Supplementary Table S1). Immediately before the first feeding on day 10, the reactors displayed similar *mcrA*/*mrtA* profiles for both reactor types, but a slight difference in the relative T-RF abundances was observed, indicating distinct acclimatization of the inoculum mixture. In Phase 1, the strictly aceticlastic genus *Methanosaeta* and the versatile genus *Methanosarcina* were more abundant in mono- than in co-digestion set-ups, whereas the strictly hydrogenotrophic genus *Methanoculleus* was more predominant in the co-digestion reactors. In both digestions, the abundance of *Methanosarcina* increased gradually from Phase 2 to Phase 6, reaching a proportion of approximately 80%. *Methanosaeta* was not detected after the acetic acid concentration started to increase in the process in Phase 4. The high affinity of *Methanosaeta* for acetate is a competitive advantage over *Methanosarcina* at low acetic acid concentrations [24], but at higher concentrations *Methanosarcina* is outcompeting *Methanosaeta*. Our observation of *Methanosaeta* at low acetic acid concentration is consistent with the findings of other studies [12,25,26]. To our surprise, at very high acetic acid concentrations during the Phase 7 and Phase 8 of mono-digestion, *Methanosaeta* was detected again, whereas the abundance of *Methanosarcina* dropped. Chen and He [27] also demonstrated competitiveness of *Methanosaeta* with *Methanosarcina* at high acetate levels. In the co-digestion reactors *Methanobacterium* predominated mainly in Phases 7 and 8. Sequences affiliated to the hydrogenotrophic genus *Methanoregula* were relatively abundant in the last phases of the experiment, when the propionate-to-acetate ratio drastically increased and the pH decreased. According to Yashiro, *et al.* [28] the genus *Methanoregula* includes acid-tolerant strains.

**Table 1:** Major reactor parameters along the eight phases of the experiment set-up.

Reactor’ Parameters	Phase 1 _(sampling Day: 19)_		Phase 2 _(sampling day: 26)_		Phase 3 _(sampling day: 33)_		Phase 4 _(sampling day: 40)_		Phase 5 _(sampling day: 55)_		Phase 6 _(sampling day: 61)_		Phase 7 _(sampling day: 68)_		Phase 8 _(sampling day: 75)_	
	Mono-	Co-	Mono-	Co-	Mono-	Co-	Mono-	Co-	Mono-	Co-	Mono-	Co-	Mono-	Co-	Mono-	Co-
	Digestion		Digestion		Digestion		Digestion		Digestion		Digestion		Digestion		Digestion	
Biogas yield * (mL·g_VS_^−1^)	1086.3	1198.2	506.9	541.0	283.9	409.6	292.8	329.2	368.3	410.9	397.6	251.2	127.9	58.3	69.7	33.7
CH_4_^§^ (%)	57.7	55.6	57.4	56.6	60.3	53.9	57.8	54.9	61.3	54.9	61.1	na	na	na	na	na
CO_2_^§^ (%)	42.3	44.4	42.6	43.4	39.7	46.1	42.2	45.1	38.7	45.1	38.9	na	na	na	na	na
Acetic acid (mg·L^−1^)	55.7	37.3	26.7	25.4	46.1	69.6	160.3	120.5	240.6	155.6	145.1	141.9	1003.0	914.2	1370.0	1334.1
Propionic acid (mg·L^−1^)	10.7	6.9	6.7	4.9	5.3	4.6	13.1	8.8	10.4	5.8	208.0	283.7	537.6	550.7	433.9	391.8
*n*-Butyric acid (mg·L^−1^)	4.7	2.5	3.1	2.0	2.2	1.8	19.5	12.1	5.3	1.8	26.0	6.7	428.9	403.3	1144.8	1193.0
VOA (g·L^−1^)	0.8	0.8	0.6	0.7	0.6	0.6	0.5	0.5	1.0	0.8	0.6	0.6	2.2	2.1	na	na
VOA/TIC (g_VOA_·g_CaCO3_^−1^)	0.2	0.2	0.2	0.1	0.2	0.2	0.2	0.2	0.2	0.2	0.7	0.6	3.1	3.1	na	na
pH *	7.5	7.5	7.4	7.3	7.5	7.5	7.2	7.1	7.2	6.9	7.0	6.5	6.3	5.7	5.4	5.2
NH_4_-N (g·L^−1^)	1.0	1.1	0.8	0.8	0.6	0.9	0.5	0.5	0.3	0.3	0.1	0.1	0.2	0.1	0.1	0.2
TS (%)	3.1	2.9	2.7	2.6	2.5	2.7	2.3	2.8	2.1	2.3	2.1	2.1	5.6	na	6.9	7.6
VS (%)	2.1	2.0	1.8	1.7	1.7	1.8	1.6	1.9	1.5	1.7	1.6	1.5	3.8	na	4.5	5.0

* Only for these parameters an average of all measurements during each specific Phase was done, since these parameters were analyzed almost every day; ^§^ Trace gases were not detected in our measurements with the applied technique, therefore we rounded our CH_4_ and CO_2_ values to 100%; “sampling day” corresponds to the last Phase day, when the samples were analyzed; “na” refers to not analysed due to technical operation problems: the very low biogas production on the last two phases hindered the GC measurement for gas composition; the low pH values detected on the last phase hindered the titration of sample for measuring VOA and VOA/TIC; and the TS and VS measurement was hindered by technical mistake while handling the samples.

### 2.3. Isotopic Changes of the Produced Biogas

Carbon-stable isotope compositions of filter cake and bagasse samples were analyzed since they influence the final isotope composition of the produced methane [5]. Filter cake and bagasse had isotope signatures of −14.30‰ and −13.64‰ δ^13^C, respectively, which are in the typical range for C4 plants (δ^13^C values between −12‰ and −16‰) [29].

**Figure 2:**
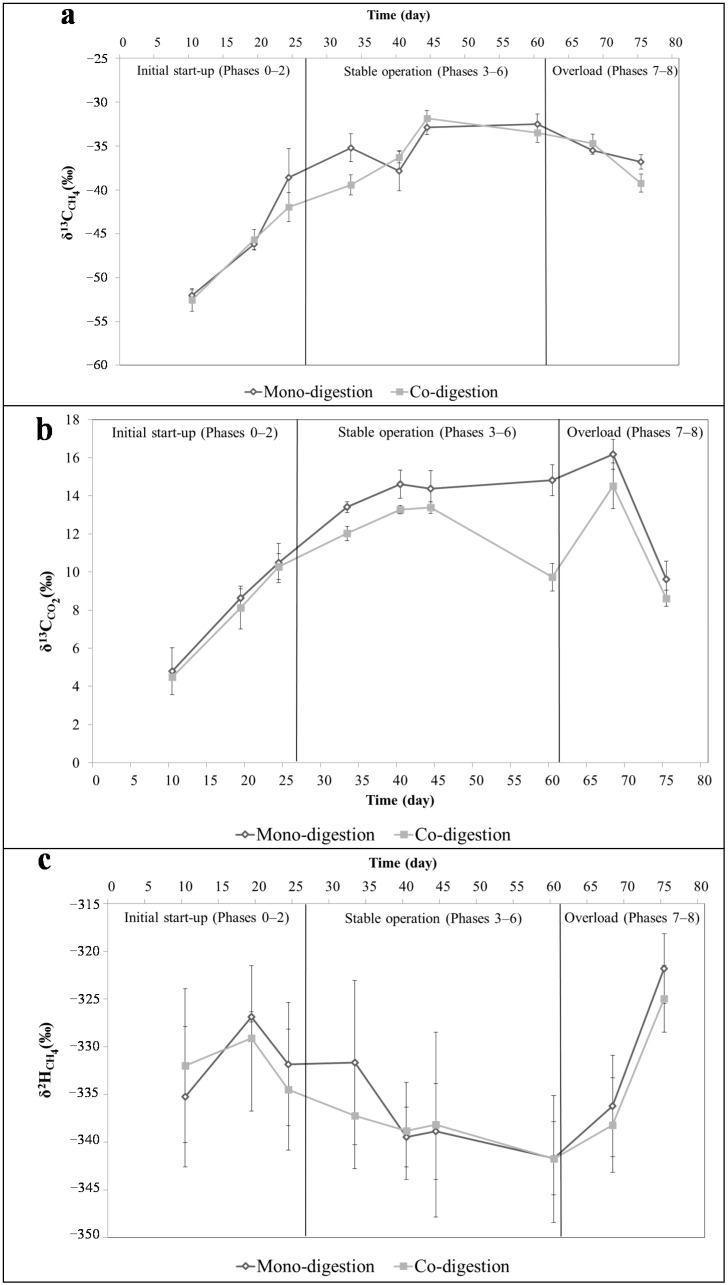
Isotopic dynamics of δ^13^C_CH4_ (**a**); δ^13^C_CO2_ (**b**); and δ^2^H_CH4_ (**c**) along gradual OLR increase in biogas reactors fed with sugarcane waste products. Isotope data of CO_2_ during the last overload phase contains data uncorrected regarding the pH shift induced degassing.

The gradual overload of the mono- and co-digestion reactors resulted in process changes that were monitored via the isotope composition of the biogas in terms of δ^13^C_CH4_, δ^13^C_CO2_, and δ^2^H_CH4_ (Figure 2). Both digestion set-ups had very similar dynamics. The δ^13^C_CH4_ became enriched from −52‰ to about −32‰ along the gradual OLR increase until Phase 5 at 2.5 g_vs_·L^−1^·day^−1^ (Figure 2a). Following, the Phase 6 had similar isotope values as the previous phase. This stationary isotope signature around −32‰ is consistent with former studies that found similar isotope fractionation of biogas samples from continuous stirred tank reactors (CSTRs) fed with C4 plant maize silage [5,19]. The inhibition of biogas production in co-digestion in Phase 6 also coincided with the isotopic depletion of the methane associated with slightly lighter δ^13^C_CH4_ values. In the last two phases of the experiment, when the process was clearly inhibited, depletion of δ^13^C_CH4_ values was observed.

The δ^13^C of carbon dioxide in the produced biogas in the mono- and co-digestion presented also similar trends (Figure 2b) with an enrichment from 4‰ to about 15‰. However, only in Phase 6 the tendency between both digestion set-ups differed. In this case, the biogas production inhibition in co-digestion may have resulted in abrupt δ^13^C_CO2_ depletion. Phase 7 had the most enriched δ^13^C_CO2_ composition, followed by drastic depletion in ^13^C values in Phase 8. However, the observed isotope effect is at certain extent due to the decreasing pH and the associated fast degassing of the CO_2_ from the bicarbonate in the liquid.

The hydrogen isotope composition of methane (δ^2^H_CH4_) in the mono- and co-digestion shows similar trends (Figure 2c). The hydrogen isotope compositions showed an opposite tendency to carbon isotope composition regarding enrichment and depletion periods. After the feeding regime has stated at Phase 1 the isotope values depleted from around −327‰ to −342‰ at the end of the stable operation phases. In the final phases, δ^2^H_CH4_ enriched to about −322‰ when OLR drove the methanogenic process to collapse.

### 2.4. Methanogenic Pathways

**Figure 3:**
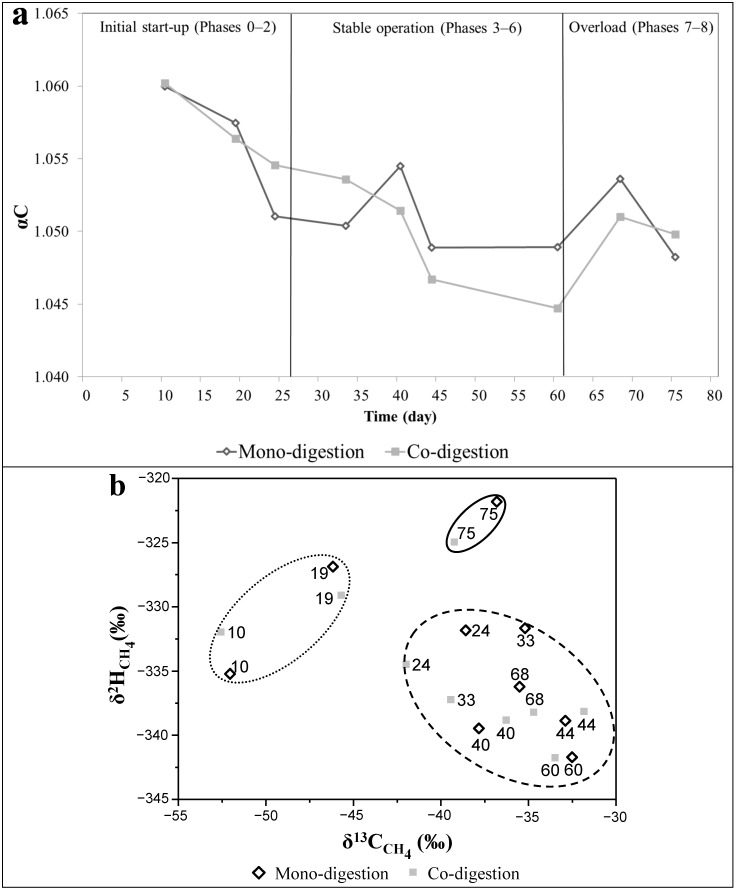
Characterization of the potential predominant methanogenic pathway along gradual OLR increase in biogas reactors fed with sugarcane waste products in mono- and co-digestion. In diagram (**a**) the dynamic shift of αC values are shown, while diagram (**b**) presents the correlation of δ^2^H_CH4_ and δ^13^C_CH4_; In (**b**) the dotted, dashed and lined hulls represent the beginning, middle, and end of the experiment, respectively. The numbers in the graphic indicate the experiment day.

The apparent fractionation factor (αC) calculated based on δ^13^C_CH4_ and δ^13^C_CO2_ composition as previously described [30,31,32] was used to identify a predominance of hydrogenotrophic and aceticlastic methanogenesis (Figure 3a). An intermediate αC value ranging between 1.065 (>for hydrogenotrophic) and 1.025 (<for aceticlastic) was found in our experiment, indicating that the methane produced during increasing OLR was derived similarly from both methanogenic pathways. This agrees with the broad spectrum of methanogenic genera (Figure 1) which included the versatile genus *Methanosarcina*, the strictly aceticlastic genus *Methanosaeta* and the strictly hydrogenotrophic order Methanomicrobiales (*Methanoculleus* and *Methanoregula*-related microorganisms) and the genus *Methanobacterium*. However, the high αC values 1.060 and 1.056 at the beginning of the experiment, in Phase 0 (at day 10 just before the first feeding) and Phase 1, respectively, indicated a predominance of hydrogenotrophic methanogenesis, suggesting that *Methanosarcina* was using this pathway together with *Methanoculleus* and *Methanobacterium*. The substrates from other AD processes in the mixture of digestate inoculated in our reactors may have also contributed to the initial isotope composition. Along the experiment, the relative abundance of *Methanosarcina* gradually increased and may have slightly shifted the methanogenesis from the hydrogenotrophic towards the aceticlastic pathway until the OLR of 3.0 g_vs_·L^−1^·day^−1^ was reached. In this case, the composition of sugarcane waste and the added water favored a tendency towards aceticlastic methanogenesis, though the strictly aceticlastic genus *Methanosaeta* was no longer abundant after Phase 4 in both digestion set-ups. However, in case of mono-digestion in the inhibition-characterized Phases 7 and 8, sequences affiliated with the genus *Methanosaeta* were detected again. A similar finding was described by Schmidt, *et al.* [33] who observed that decreasing HRT may favor the genus *Methanosaeta* under certain conditions. Nikolausz, *et al.* [5] described that more depleted δ^13^C_CH4_ values in biogas reactors potentially indicate a shift toward the dominance of hydrogenotrophic methanogenesis. This observation was also supported by our results. Since the first feeding with the sugarcane waste products *Methanobacterium* became abundant with minor changes along mono-digestion and with increase in dominance during overload of co-digestion reactors. This was shown by the increase of the αC values at reactor overload, which also indicated a shift towards hydrogenotrophic methanogenesis. In addition, the relative abundance of the other hydrogenotrophic taxon related to the genus *Methanoregula* also increased during reactor overload in both digestion set-ups.

The combination plot of δ^13^C_CH4_ and δ^2^H_CH4_ as function of increasing ORL is shown in Figure 3b, where the dotted, dashed, and lined hulls represent the beginning, middle, and end of the experiment, respectively. Phases 0 and 1 are represented in the dotted hull with higher αC values as described earlier. The dashed hull area is covering most of the phases (from Phase 2 to Phase 7), which had similar ranges and trends of αC values for mono-digestion (1.049–1.055) and co-digestion (1.045–1.055). Samples from Phase 8 are grouped into the lined hull, representing the period when the mono- and co-digestion reactors were overloaded, imbalances were clearly observed and less depleted δD values were measured. The isotope effect associated with aceticlastic methanogenesis is significantly larger in case of hydrogen derived from the water, but it affects only one out of four hydrogen atoms of the methane, while the other three atoms are influenced by the δD of the organic matter. In natural environments aceticlastic methanogenesis results in more depleted δD values of methane, which is in agreement with our data where depleted values were observed during the stable reactor operating conditions and explained by the predominance of *Methanosarcina*.

**Figure 4:**
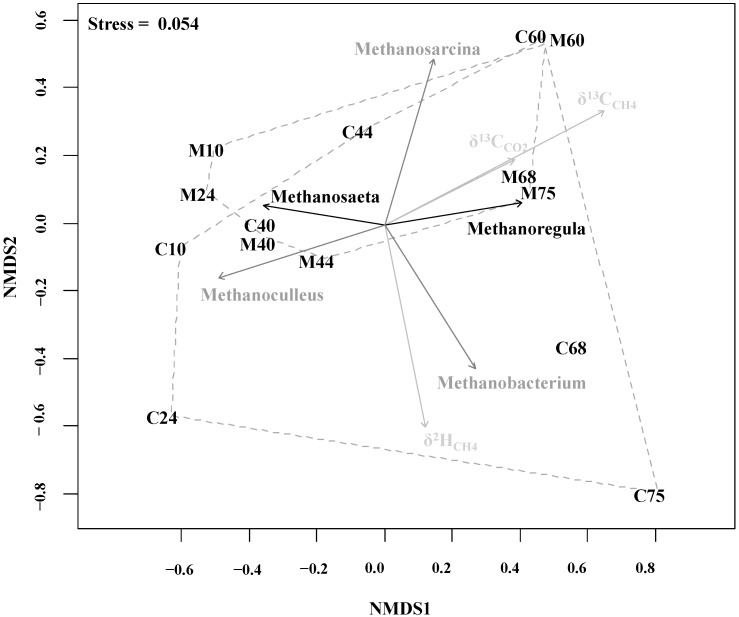
NMDS analysis plot for correlating the T-RFLP profile of methanogens with the isotope composition of produced biogas. The smaller and bigger hull in the diagram represents the mono- and co-digestion in several sampling time, respectively. The letter M stands for mono-digestion and C for co-digestion set-up and the following numbers correspond to the sampling day. The dim grey and black arrows indicate the highly significant (*p* < 0.001) and significant (*p* < 0.05) correlations, respectively. Grey arrows indicate the correlation vectors of community differences and the isotope composition at lower significance (*p* < 0.5). Monte-Carlo permutation was used to test the significance against 999 random data sets. The direction of the arrows show the correspondence to the community structures and the length of the arrow indicate the strength of the correlation with the ordination axis.

In Figure 4 the correlation between the T-RFLP profile dynamics and the isotope composition of biogas is shown in a non-metric multidimensional scaling (NMDS) plot. The methanogenic pathway shift can be viewed in the NMDS plot as shifts of T-RFLP profile clusters (dashed hulls) during the different phases of the ORL increase. The methanogenic community most significantly correlates with the isotopic fractionation of δ^13^C_CH4_ as indicated by the vector converted to the larger grey arrow in the NMDS plot. The reactor overload in Phases 7 and 8 for mono-digestion was characterized by a strong correlation of the hydrogenotrophic taxon related to the genus *Methanoregula* and less depleted isotopic values of δ^13^C_CH4_ and δ^13^C_CO2_, whereas for co-digestion it was characterized by a significant correlation of the strict hydrogenotrophic genus *Methanobacterium* and δ^2^H_CH4_. This corroborates the increase of the αC values for both digestion set-ups at reactor overload in Phase 7 indicating the shift towards hydrogenotrophic methanogenesis and higher αC values of co-digestion compared to mono-digestion at the Phase 8, when the relative abundance of *Methanobacterium* was around 75%.

## 3. Experimental Section

### 3.1. Biogas Reactors, Operation and Analytical Methods

The experiment was carried out in four CSTRs under mesophilic conditions at 38 °C. In order to provide a diverse microbial start-up community as inoculum, each reactor was inoculated with a mixture of digestates from several CSTRs, which had been fed daily with either maize silage, dried distillers grains with soluble (DDGS), straw or chicken manure. In our experiment, filter cake and bagasse, two solid waste products from the bioethanol industry (Goiás, Brazil), were used as substrate. Bagasse was cut to 1 mm pieces by milling to increase the accessible surface area and to facilitate reactor feeding and stirring. The TS and VS were 28% and 17% for filter cake and 57% and 55% for bagasse, respectively. Mono-digestion was performed with filter cake, whereas co-digestion reactors were fed at a substrate ratio of 70% filter cake and 30% bagasse (based on fresh mass), corresponding to a VS-based filter cake to bagasse ratio of 1:0.74. All four reactors were fed every day according to the digestion set-up.

Table 2 shows further technical parameters of the operation. The experiment was divided into eight phases according to the gradual increase of the OLR. Biogas production was monitored, by counting biogas bubbles in a liquid-filled pipe via digital imaging and size recognition [34]. Biogas composition was measured with a thermal conductivity detector Chrompack Micro GC CP-2002P (Middelburg, The Nederland). The TS and VS, the pH values, the NH_4_-N concentration, acetate, propionate, and *n*-butyrate were determined as described previously by Leite, *et al.* [1]. The total VOA concentration and the VOA/TIC were analyzed as described earlier by Ziganshin, *et al.* [35].

### 3.2. Methanogenic Community Analysis

Duplicate digestate samples were collected in 2-mL test tubes and immediately stored at −20 °C for further analysis. Total DNA isolation was carried out using NucleoSpin^®^ Soil kit (Macherey-Nagel, Düren, Germany). Methanogen-specific methyl coenzyme-M reductase (*mcrA*) gene fragments were amplified by polymerase chain reaction (PCR) using the forward primer mlas and the reverse primer mcrA-rev labeled with 6-carboxyfluorescein (FAM) for T-RFLP analyses. Non-labeled primers were used for molecular cloning and sequencing as in a previous study [5]. Further, T-RFLP screening and partial sequencing of purified PCR products were performed as described by Nikolausz, *et al.* [5]. The BLASTN and BLASTX tools were used to search for similar sequences in public databases. The *mcrA*/*mrtA* gene sequences obtained in this study were deposited in the European Bioinformatics Institute (EMBL-EBI) database under the accession numbers LN847074-LN847091. The NMDS analyses were conducted as described by Sträuber, *et al.* [36].

**Table 2:** Technical parameters during the experiment set-up of the mono- and co-digestion of filter cake and bagasse.

Set-up-Technical Parameters	Phase 1		Phase 2		Phase 3		Phase 4		Phase 5		Phase 6		Phase 7		Phase 8	
	Mono-	Co-	Mono-	Co-	Mono-	Co-	Mono-	Co-	Mono-	Co-	Mono-	Co-	Mono-	Co-	Mono-	Co-
	Digestion		Digestion		Digestion		Digestion		Digestion		Digestion		Digestion		Digestion	
Experiment phase (day)	11–19		20–26		27–33		34–40		41–55		56–61		62–68		69–75	
Substrate (g _fresh mass_)	2.4	1.5	4.9	3.0	7.3	4.5	9.7	6.0	12.2	7.5	14.6	9.0	10.7	10.5	12.2	12.0
Water mixed with substrate (mL)	20		25		30		35		45		45		50		55	
Working volume (L)	0.8		0.8		0.8		0.8		0.8		0.8		0.5	0.8	0.5	0.8
VS (g·day^−1^)	0.4		0.8		1.2		1.6		2.0		2.4		1.8	2.8	2.0	3.2
OLR (g_VS_·L^−1^·day^−1^)	0.5		1.0		1.5		2.0		2.5		3.0		3.5		4.0	
HRT (day)	35.7	37.2	26.8	28.6	21.4	23.2	17.9	19.5	15.3	15.2	13.4	14.8	8.2	13.2	7.4	11.9

### 3.3. Stable Isotope Analysis

The carbon isotope composition of the solid waste products (filter cake and bagasse) were measured in a continuous flow system consisting of an elemental analyser (Euro EA, HEKAtech GmbH, Wegberg, Germany) connected to an isotope ratio mass spectrometer (Finnigan MAT 253, Thermofinnigan, Bremen, Germany).

Biogas from the reactor headspace was sampled with a syringe at the same time as digestate was sampled. Twenty mL biogas was transferred and stored in gas-tight pre-evacuated vials until further analysis. Isotope measurements were performed as described by Feisthauer, *et al.* [37]. Briefly, an isotope ratio mass spectrometry system (Finnigan MAT 253, Thermofinnigan, Bremen, Germany) was coupled to a gas chromatograph (HP 6890 Series, Agilent Technology, Santa Clara, CA, USA) either via a combustion device for carbon analysis or via a pyrolysis unit for hydrogen analysis. Fifty μL of biogas sample from the vials were injected into a helium carrying tube at the split ratio of 1:50 for carbon and 1:5 for hydrogen analysis.

## 4. Conclusions

Strong dynamics of community structure and pathway shifts in methanogens were observed by molecular and stable isotope fingerprinting during gradual increase of OLR. The overloading effect in both digestion set-ups was observed beginning at an OLR of 2.5 g_vs_·L^−1^·day^−1^ from the increase of the propionate-to-acetate ratio. However, the co-digestion processes suffered process failure earlier (at OLR 3.0) than mono-digestion (at OLR 3.5). Until process-overload *Methanosarcina* became gradually predominant, shifting the methanogenic pathway towards aceticlastic. The change towards hydrogenotrophic methanogenesis during reactor overload might be taken as an indicator for process failure. Monitoring of the methanogenic pathways by stable isotope composition of biogas can be an excellent tool to control and predict process failure.

## Supplementary Materials

Supplementary materials can be found at http://www.mdpi.com/1422-0067/16/10/23210/s1.
