# Supplementary material for: Improved Monitoring of Semi-Continuous Anaerobic Digestion of Sugarcane Waste: Effects of Increasing Organic Loading Rate on Methanogenic Community Dynamics
Source: Int J Mol Sci. 2015 Sep 25;16(10):23210–26. doi: 10.3390/ijms161023210 (PMC4632694; doi:10.3390/ijms161023210)
Supplement: Supplementary file 1 [file ijms-16-23210-s001.pdf]

Supplementary Information

Table S1. Sequence analyses of representative *mcrA/mrtA* gene clones.

Clone (Acc. No.)	Sequences	Closest Cultivable Relative (Acc. No.) with Sequence Identity	BstNI T-RF (bp)
Methanobacterium	-	-	-
E10-8H2 (LN847074)	*4*	*Methanobacterium congolense* strain NBRC 105227 (AB542748.1) 73%	469
E2-8H1 (LN847075)	*6*	*Methanobacterium formicicum* (LN515531.1) 86%	470
C9-8C1 (LN847076)	*6*	*Methanobacterium formicicum* strain BRM9 (CP006933.1) 86%	463
C1-8H2 (LN847077)	*8*	*Methanobacterium kanagiense* (AB551869.1) 90%	469
E8-8H1 (LN847078)	*3*	*Methanobacterium sp.* MB1 (HG425166.1) 99%	332
Methanoculleus	-	-	-
A1-8C1(LN847079)	*6*	*Methanoculleus bourgensis* MS2T (HE964772.2) 99%	93
H8-8C2 (LN847080)	*1*	*Methanoculleus bourgensis* strain MAB1 (KJ708788.1) 93%	93
C6-8C2 (LN847081)	*9*	*Methanoculleus chikugoensis* (AB288270.1) 93%	91
D10-8C2 (LN847082)	*1*	*Methanoculleus chikugoensis* strain NBRC 101202 (AB703634.1) 90%	92
A12-8C1 (LN847083)	*6*	*Methanoculleus sp.* M07 (AB288284.1) 93%	92
Methanomassiliicoccus	-	-	-
C10-8C1 (LN847084)	*2*	*Methanomassiliicoccus luminyensis* strain B10 (HQ896500.1) 93%	408
Methanoregula	-	-	
H3-8H2 (LN847085)	*6*	*Methanoregula formicicum* SMSP (CP003167.1) related 81%	338
Methanosaeta	-	-	-
A10-8C1(LN847086)	*15*	*Methanosaeta concilii* strain NBRC 103675 (AB679170.1) 93%	127
Methanosarcina	-	-	-
A4-8C1 (LN847087)	*9*	*Methanosarcina mazei* Tuc01 (CP004144.1) 93%	55
B3-8C1 (LN847088)	*29*	*Methanosarcina thermophila* TM-1 (AB353225.1) 96%	56
B1-8XH1 (LN847089)	*1*	*Methanosarcina mazei* strain KOR-4 (KC292223.1) 99%	55
B9-8XH1 (LN847090)	*1*	*Methanosarcina mazei* strain NBRC 101201 (AB703645.1) 99%	487
Methanospirillum	-	-	-
B11-8C1 (LN847091)	*3*	*Methanospirillum stamsii* strain Pt1 (KC951357.1) 95%	344
